# Psychological distress in the academic population and its association with socio-demographic and lifestyle characteristics during COVID-19 pandemic lockdown: Results from a large multicenter Italian study

**DOI:** 10.1371/journal.pone.0248370

**Published:** 2021-03-10

**Authors:** Marco Fornili, Davide Petri, Carmen Berrocal, Giuseppe Fiorentino, Fulvio Ricceri, Alessandra Macciotta, Andreina Bruno, Domenica Farinella, Michela Baccini, Gianluca Severi, Laura Baglietto

**Affiliations:** 1 Department of Clinical and Experimental Medicine, University of Pisa, Pisa, Italy; 2 Department of Surgical, Medical and Molecular Pathology, and Critical Care Medicine, University of Pisa, Pisa, Italy; 3 Italian Naval Academy, Livorno, Italy; 4 Department of Clinical and Biological Science, University of Turin, Turin, Italy; 5 Department of Education Science, University of Genoa, Genoa, Italy; 6 Department of Political Science and Law, University of Messina, Messina, Italy; 7 Department of Statistics, Informatics, Applications “Giuseppe Parenti”, University of Florence, Florence, Italy; 8 CESP, Fac. de Médecine—Univ. Paris-Sud, Fac. de Médecine—UVSQ, INSERM, Université Paris-Saclay, Villejuif, France; 9 Gustave Roussy, Villejuif, France; National Institute for Infectious Diseases Lazzaro Spallanzani-IRCCS, ITALY

## Abstract

Measures implemented in many countries to contain the COVID-19 pandemic resulted in a change in lifestyle with unpredictable consequences on physical and mental health. We aimed at identifying the variables associated with psychological distress during the lockdown between April and May 2020 in the Italian academic population. We conducted a multicenter cross-sectional online survey (IO CONTO 2020) within five Italian universities. Among about 240,000 individuals invited to participate through institutional communications, 18 120 filled the questionnaire. Psychological distress was measured by the self-administered Hospital Anxiety and Depression Scale (HADS). The covariates collected included demographic and lifestyle characteristics, trust in government, doctors and scientists. Associations of covariates with influenza-like symptoms or positive COVID-19 test and with psychological distress were assessed by multiple regression models at the local level; a meta-analysis of the results was then performed. Severe levels of anxiety or depression were reported by 20% of the sample and were associated with being a student or having a lower income, irrespective of their health condition and worries about contracting the virus. The probability of being severely anxious or depressed also depended on physical activity: compared to those never exercising, the highest OR being for those who stopped during lockdown (1.53; 95% CI, 1.28 to 1.84) and the lowest for those who continued (0.78; 95% CI, 0.64 to 0.95). Up to 21% of severe cases of anxiety or depression might have been avoided if during lockdown participants had continued to exercise as before. Socioeconomic insecurity contributes to increase mental problems related to the COVID-19 pandemic and to the measures to contain it. Maintaining or introducing an adequate level of physical activity is likely to mitigate such detrimental effects. Promoting safe practice of physical activity should remain a public health priority to reduce health risks during the pandemic.

## Introduction

In March 2020 in Italy, a nationwide quarantine was introduced as a measure to contain the transmission of the COVID-19 pandemic. The measure caused a radical change in the lifestyle of individuals, with unpredictable consequences on their mental and physical health. The impact that isolation and social distancing can have on lifestyle habits and their consequences on the levels of anxiety and depression has been studied in very specific contexts [1]. During the lockdown periods, stress, forced physical inactivity and lifestyle changes can produce changes in metabolic parameters and obesity, leading to an increase in risk of cardiovascular events [2], post-traumatic stress disorder and depressive symptomatology [3–5]. Little is known about the effect of quarantine on the academic community, including students and employees, that in the context of the COVID-19 pandemic did not represent a group at high risk of severe health consequences. However, given the high incidence of emotional disorders among university students, the impact of lockdown measures on their mental health can be particularly relevant [6, 7]. A few studies investigated the effect of COVID-19 pandemic on mental health of student during confinement [8–11]. A nationwide survey conducted in France among more than 69,000 university students reported prevalences of 27% and 16% for anxiety and depression respectively [12]. A study conducted during the COVID-19 lockdown on 2,530 students and workers of a Spanish university found that 21%, 34% and 28% of the participants suffered from anxiety, depression and stress, respectively [13]. Fear of becoming infected, worries for friends and relatives, economic uncertainty, loneliness can all be determinants of increased anxiety and depression during lockdown [5]. Other factors that can contribute to increase emotional distress are prolonged physical inactivity and other changes in lifestyle and working routine [14, 15]; the effects of physical activity on stress and anxiety during the COVID-19 pandemic have already been discussed in literature [16].

The aim of our study was to measure the level of psychological distress during the lockdown period in response to the COVID-19 pandemic and investigate its association with socio-demographic characteristics and lifestyle in a large sample of the Italian academic population.

## Methods

### Study design

A large cross-sectional online survey was conducted in five Italian universities between April and May 2020. The study was approved by the Committee on Bioethics of the University of Pisa (Review No. 10/2020, 3^rd^ of April 2020). Participants provided informed consent before completing the questionnaire. The survey included sections designed to assess socio-demographic characteristics, health status, psychological distress and changes related to work and lifestyle in the academic population during the nationwide COVID-19 lockdown that started on the 10^th^ of March 2020. Data were collected through the Moodle learning platform of each university, anonymized and analyzed at the local level. Then the results from each participating center were sent to the Unit of Medical Statistics of the University of Pisa where a meta-analysis was conducted.

### Study population

Among about 220,000 students and 20,000 employees of the Italian universities of Turin and Genoa (Northern Italy), Florence and Pisa (Central Italy) and Messina (Southern Italy) invited to participate in the online survey, 6% of the students and 19% of the employees filled the questionnaire. After excluding 1,046 participants who did not fill the section on psychological distress, 17,074 were left for the analysis.

### Measurements and covariates

Students and employees of the participating universities have been invited to fill the questionnaire through institutional communications. The survey took 20 to 30 minutes to be completed. The structured online survey included questions about socio-demographic characteristics of participants, housing, habits and symptoms related to COVID-19 and lifestyle during quarantine. Information about general health status of participants was collected (questions about 12 chronic conditions were asked) together with their influenza-like symptoms (temperature, cough or respiratory problems) or diagnosis of COVID-19 during quarantine. Psychological distress, one of the main indicators of mental health, was assessed through the Italian version of the Hospital Anxiety and Depression Scale (HADS) [17–19], a validated instrument composed by 7 items related to anxiety and 7 items related to depression. Each item is rated on a 4-score Likert scale ranging from 0 to 3, resulting in a total score ranging from 0 to 21 in each of the two components. On both subscales of anxiety and depression, subjects with scores ≤7, 8–10 and ≥11 were classified as normal, borderline cases or severe cases, respectively [20].

### Statistical analysis

Data collected at each participating center were anonymized for statistical analysis. Categorical variables were described as absolute frequencies and percentages. The heterogeneity of the distribution of each variable across universities was assessed by Fisher’s exact test. The associations of covariates with influenza-like symptoms or positive COVID-19 test and HADS scores were assessed by logistic and linear regression models, respectively. For both analyses two models were fitted: one including each covariate at a time together with age and sex (Model 1); the other including all covariates, except education level in the family and cohabitants (Model 2). Multinomial logistic regressions were fitted to estimate the odds ratios (ORs) of borderline versus normal and severe versus normal levels of anxiety and depression for categories of physical activity; logistic regressions were fitted to estimate the ORs of either severe level of anxiety or depression versus normal level of anxiety and depression; regression models were adjusted for all covariates. The number of severe cases of anxiety and depression predicted by the logistic models were calculated under the observed distribution of physical activity during lockdown and under the hypothetical scenario where the level of physical activity during lockdown were the same as before; then the percentage of severe cases that would have been avoided by continuing to exercise were calculated. The corresponding standard errors were estimated through a non-parametric bootstrap.

First, we fitted separate models for each participating center; then, we combined the parameters estimated from the models–i.e. linear coefficients and estimated marginal means (EMMs) from the linear models; ORs and percent reduction of the number of severe cases from the logistic regression models—through a random effect meta-analysis.

All statistical analyses were conducted with the R statistical software, version 4.0.2, and all tests were two-sided at the 0.05 significance level.

## Results

Among the 18,120 participants 9,062 (50%) were from universities in Northern Italy, 8,264 (46%) from Central Italy and 794 (4%) from Southern Italy. Socio-demographic and lifestyle characteristics of participants are reported in Table 1. Sixty-seven percent of the participants, 67% were females and 78% were students. Overall, 23% of participants reported having suffered influenza-like symptoms (temperature, cough, breathing problems).

**Table 1:** General characteristics of participants.

	All		Turin	Genoa	Florence	Pisa	Messina	*P-value*
Variable	N	%	%	%	%	%	%	
**Age categories (years)**								*<0*.*001*
<30	13,043	72.4	76.9	71.2	70.7	74.5	45.6	
30–39	1,413	7.8	7.3	8.3	7.8	7.7	9.5	
40+	3,555	19.7	15.7	20.5	21.4	17.8	44.9	
**Gender**								
Female	11,980	67.2	73.6	66.8	68.0	62.5	63.3	*<0*.*001*
Male	5,849	32.8	26.4	33.2	32.0	37.5	36.7	
**Position**								
Student	14,028	78.3	80.4	78.1	77.8	80.3	54.1	*<0*.*001*
Technical/administrative staff	1,810	10.1	8.8	9.8	11.1	8.8	24.7	
Teaching/research staff	2,088	11.6	10.8	12.1	11.0	10.9	21.1	
**Household monthly income**								
Low (≤1500 euros)	3,619	24.7	24.5	24.5	22.7	25.6	28.1	*0*.*003*
Medium (between 1501 and 3000 euros)	6,342	43.3	43.4	43.9	43.2	43.8	36.8	
High (>3000 euros)	4,684	32.0	32.1	31.5	34.1	30.6	35.1	
**Education family (Which is the highest level of education within your nuclear family?)**								
Primary	453	2.6	2.8	2.2	2.9	2.4	3.1	*<0*.*001*
Secondary	5,734	32.3	34.4	33.0	31.6	31.2	27.0	
University degree	7,362	41.5	42.5	41.7	41.2	41.4	36.8	
Master degree	1,048	5.9	5.9	6.0	4.9	6.6	4.4	
PhD or equivalent	3,138	17.7	14.4	17.1	19.4	18.4	28.7	
**House with a garden or balcony**								
No	2,125	11.9	8.7	15.7	10.8	12.6	7.8	*<0*.*001*
Yes	15,664	88.1	91.3	84.3	89.2	87.4	92.2	
**Cohabitants**								
No	976	5.5	5.4	5.4	4.8	5.8	6.4	*0*.*35*
Yes	16,871	94.5	94.6	94.6	95.2	94.2	93.6	
**Old or disabled (Do you live with old or disabled people?)**								
No	14,138	84.7	85.4	84.2	85.0	85.3	78.3	*<0*.*001*
Yes	2,555	15.3	14.6	15.8	15.0	14.7	21.7	
**Currently working with the public (Are you currently working with the public?)**								
No	17,254	95.5	95.1	94.8	95.5	96.7	94.1	*<0*.*001*
Yes	805	4.5	4.9	5.2	4.5	3.3	5.9	
**Cohabitants currently working with the public (Are your cohabitants currently working with the public?)**								
No	10,154	58.0	51.3	56.4	56.1	64.7	64.6	*<0*.*001*
Yes	7,345	42.0	48.7	43.6	43.9	35.3	35.4	
**General health (number of reported existing health conditions)**								
0	12,538	69.2	68.8	69.0	69.4	70.9	60.1	*<0*.*001*
1	3,838	21.2	22.1	21.1	20.8	20.6	22.3	
2+	1,744	9.6	9.1	10.0	9.7	8.6	17.6	
**Symptoms (Since the beginning of the quarantine have you been diagnosed with COVID-19 or did you suffer of any of the following symptoms: fever, cough, respiratory problems)**								
No	13,499	77.3	75.1	75.9	79.6	78.2	84.9	*<0*.*001*
Yes	3,954	22.7	24.9	24.1	20.4	21.8	15.1	
**Worries (Are you worried to contract the virus?)**								
No	12,238	78.8	78.9	75.2	81.5	79.7	83.6	*<0*.*001*
Yes	3,291	21.2	21.1	24.8	18.5	20.3	16.4	
**Adequacy of the measures (How do you judge the measures adopted by the government to contain the spreading of the infection?)**								
Adequate	7,351	42.4	36.5	39.2	46.2	45.8	56.3	*<0*.*001*
Insufficient	8,971	51.7	57.4	54.5	46.4	49.2	39.3	
Excessive	1,035	6.0	6.1	6.3	7.4	5.1	4.5	
**Trust in doctors (Do you trust medical doctors?)**								
No	778	4.4	3.3	4.6	4.8	3.8	11.1	*<0*.*001*
Yes	17,100	95.6	96.7	95.4	95.2	96.2	88.9	
**Trust in scientists (Do you trust scientists?)**								
No	742	4.2	3.0	4.8	4.8	3.3	11.3	*<0*.*001*
Yes	17,077	95.8	97.0	95.2	95.2	96.7	88.7	
**Trust in the government (Do you trust the governments?)**								
No	5,791	32.5	30.6	36.4	31.4	29.4	46.1	*<0*.*001*
Yes	12,011	67.5	69.4	63.6	68.6	70.6	53.9	
**Physical activity during lockdown (hours per week)**								
<1h	6,914	38.5	38.1	36.6	36.5	39.0	56.4	*< 0*.*001*
1-2h	3,991	22.2	21.6	21.1	22.6	23.3	23.7	
3-4h	3,777	21.0	21.2	21.9	21.4	21.4	11.6	
>4h	3,266	18.2	19.2	20.4	19.4	16.3	8.3	
**HADS-anxiety categories**								
Normal	11,301	68.6	65	70.4	69.1	70.2	65.4	*<0*.*001*
Borderline	2,675	16.2	17.2	15.3	16.7	15.8	17.4	
Severe	2,501	15.2	17.8	14.3	14.3	13.9	17.1	
**HADS-depression categories**								
Normal	11,989	78.6	78.4	78.9	77.4	79.4	75.8	*0*.*33*
Borderline	2,099	13.8	14.1	13.2	14.3	13.3	16.1	
Severe	1,168	7.7	7.5	7.9	8.3	7.2	8.1	
**HADS categories**								
Normal	11,997	80.3	78.6	80.6	81.2	81.4	77.5	*0*.*006*
Severe anxiety or severe depression	2,945	19.7	21.4	19.4	18.8	18.6	22.5	

The odds of having suffered from influenza-like symptoms were higher in younger subjects (OR for 10-year increase of age, 0.87; 95% CI, 0.80 to 0.95), in males than in females (OR, 1.19; 95% CI, 1.02 to 1.38), in individuals with comorbidities (OR for 1 versus no comorbidities, 1.71; 95% CI, 1.54 to 1.91; OR for 2 or more versus no comorbidities, 2.19; 95% CI, 1.73 to 2.77), in those worried to contract the virus (OR, 1.23; 95% CI, 1.06 to 1.42), in those judging the measures by the government not adequate (OR for insufficient versus adequate measures, 1.31; 95% CI, 1.11 to 1.53) and in individuals with lower levels of physical activity during lockdown (OR for >4 versus <1 hours per week, 0.71; 95% CI, 0.62 to 0.81; S1 Table).

The HADS questionnaire was filled by 17, 074 (94%) participants; for anxiety, borderline and severe levels were reported by 16% and 15% of respondents; for depression, 14% and 8%; these figures resulted in 20% of participants being either severely anxious or severely depressed. The results of the linear association between overall psychological distress and socio-demographic and lifestyle variables are reported in Table 2. All variables, with the exception of the presence of cohabitants, were significantly associated with the psychological distress both in the univariate and in the multiple model. Younger individuals, females and students had higher levels of psychological distress. Having a low income or low level of education in the family, both proxies of the socio-economic status, were related to higher levels of psychological distress; similar results were observed for sharing the house with old or disabled people. Working in contact with the public was associated with lower HADS levels, whereas having cohabitants working with the public was associated with higher levels. Also, higher HADS levels were associated with having a frail health, having experienced COVID-like symptoms, and being worried to become infected; on the contrary, lower HADS levels were associated with the belief that the measures adopted were adequate and with trust in doctors and institutions.

**Table 2:** Linear regression of HADS.

	Model 1			Model 2		
Variable	Categorical	Pseudocontinuous		Categorical	Pseudocontinuous	
	Coefficient (95% CI)	Coefficient (95% CI)	*P-value*	Coefficient (95% CI)	Coefficient (95% CI)	*P-value*
**Age (10-year increase)**	-0.90 (-1.00 to -0.80)		*< 0*.*001*	-1.18 (-1.37 to -0.98)		*< 0*.*001*
**Gender**						
Female	Ref		*< 0*.*001*	Ref		*< 0*.*001*
Male	-2.66 (-2.91 to -2.42)			-2.42 (-2.71 to -2.12)		
**Position**						
Student	Ref	-0.79 (-1.04 to -0.54)	*< 0*.*001*	Ref	-0.32 (-0.61 to -0.03)	*0*.*03*
Technical/administrative staff	-0.91 (-1.53 to -0.29)			-0.53 (-1.21 to 0.15)		
Teaching/research staff	-1.61 (-2.12 to -1.09)			-0.70 (-1.29 to -0.11)		
**Income**						
Low	Ref	-0.74 (-0.91 to -0.57)	*< 0*.*001*	Ref	-0.28 (-0.47 to -0.09)	*0*.*004*
Medium	-0.92 (-1.23 to -0.60)			-0.22 (-0.57 to 0.12)		
High	-1.50 (-1.84 to -1.16)			-0.55 (-0.93 to -0.17)		
**Education level in the family**						
Primary	Ref	-0.31 (-0.45 to -0.18)	*< 0*.*001*			
Secondary	-0.69 (-1.76 to 0.38)					
University degree	-1.16 (-2.20 to -0.12)					
Master degree	-0.79 (-2.12 to 0.55)					
PhD or equivalent	-1.67 (-2.96 to -0.38)					
**House with a garden or balcony**						
No	Ref		*< 0*.*001*	Ref		*< 0*.*001*
Yes	-1.63 (-2.12 to -1.14)			-1.34 (-1.77 to -0.90)		
**Cohabitants**						
No	Ref		*0*.*80*			
Yes	-0.12 (-1.08 to 0.84)					
**Old or disabled cohabitants**						
No	Ref		*< 0*.*001*	Ref		*0*.*009*
Yes	0.91 (0.54 to 1.29)			0.50 (0.12 to 0.87)		
**Currently working with the public**						
No	Ref		*< 0*.*001*	Ref		*0*.*004*
Yes	-1.03 (-1.57 to -0.48)			-1.25 (-2.12 to -0.39)		
**Cohabitants currently working with the public**						
No	Ref		*0*.*004*	Ref		*< 0*.*001*
Yes	0.35 (0.11 to 0.59)			0.51 (0.23 to 0.80)		
**General health (number of comorbidities)**						
0	Ref	2.26 (1.84 to 2.69)	*< 0*.*001*	Ref	1.82 (1.34 to 2.30)	*< 0*.*001*
1	2.14 (1.62 to 2.66)			1.91 (1.24 to 2.59)		
2+	4.62 (3.78 to 5.47)			3.55 (2.74 to 4.37)		
**Symptoms**						
No	Ref		*< 0*.*001*	Ref		*< 0*.*001*
Yes	1.95 (1.64 to 2.25)			1.25 (0.88 to 1.63)		
**Worries**						
No	Ref		*< 0*.*001*	Ref		*< 0*.*001*
Yes	2.80 (2.50 to 3.10)			2.35 (2.02 to 2.69)		
**Adequacy of the measures**						
Adequate	Ref	1.64 (1.43 to 1.85)	*< 0*.*001*	Ref	1.14 (0.90 to 1.37)	*< 0*.*001*
Insufficient	1.64 (1.41 to 1.88)			1.05 (0.76 to 1.34)		
Excessive	3.22 (2.52 to 3.92)			2.42 (1.59 to 3.25)		
**Trust in doctors**						
No	Ref		*< 0*.*001*			
Yes	-3.38 (-4.11 to -2.64)					
**Trust in scientists**						
No	Ref		*< 0*.*001*			
Yes	-2.65 (-3.49 to -1.82)					
**Trust in the government**						
No	Ref		*< 0*.*001*			
Yes	-1.85 (-2.18 to -1.53)					
**Trust in doctors, scientists and the government**						
No	Ref		*< 0*.*001*	Ref		*< 0*.*001*
Yes	-1.89 (-2.23 to -1.54)			-1.32 (-1.73 to -0.92)		
**Physical activity during quarantine**						
<1h	Ref	-0.93 (-1.03 to -0.83)	*< 0*.*001*	Ref	-0.86 (-0.98 to -0.74)	*< 0*.*001*
1-2h	-1.49 (-1.80 to -1.19)			-1.30 (-1.66 to -0.94)		
3-4h	-2.43 (-2.74 to -2.12)			-2.09 (-2.46 to -1.71)		
>4h	-2.60 (-2.93 to -2.26)			-2.46 (-2.85 to -2.07)		

Model 1: linear regression adjusted for age and sex; Model 2: linear regression adjusted for age and sex and all variables in the table. Model 2 is based on the subsample of 9,091 participants with no missing value in any of the variables in the table.

HADS scores significantly decreased with levels of physical activity during quarantine and the association was not confounded by any of the variables we were able to adjust for. In particular, compared with those who did not exercised during quarantine, those reporting the highest level of physical activity had on average a HADS score 2.46 points lower (95% CI, 2.07 to 2.85; Table 2). In order to account for the effect of physical activity before quarantine, we fitted a model with both variables and their interaction. According to this model, those who performed physical activity before quarantine and then stopped to exercise had higher HADS scores than those who never exercised (EMM, 14.5; 95% CI, 14.2 to 14.8, versus 13.0; 95% CI, 12.6 to 13.4). Physical activity during quarantine was inversely associated with HADS scores (p < 0.001) irrespective of the physical activity status before quarantine, but its effects were more pronounced among those who exercised before quarantine than among those who did not: the estimated differences of the HADS score between those with the highest versus lowest level of physical activity were -1.8 (95% CI, -2.8 to -0.8) and -3.0% (95% CI, -3.4 to -2.5) in the group who did not exercised before lockdown and in those who did, respectively. As a result, HADS scores in those with the highest level of physical activity were very similar in the two groups (Table 3).

**Table 3:** HADS estimated marginal means (EMMs) by levels of physical activity.

	Physical activity before lockdown	
	Less than one hour per week	At least one our per week
	EMM (95% CI)	EMM (95% CI)
**Physical activity during lockdown**		
Less than 1 hour per week	13.0 (12.6 to 13.4)	14.5 (14.2 to 14.8)
1–2 hours per week	12.4 (11.9 to 13.0)	12.6 (12.2 to 13.0)
3–4 hours per week	11.6 (11.0 to 12.2)	11.8 (11.5 to 12.2)
More than 4 hours per week	11.2 (10.1 to 12.4)	11.5 (11.1 to 11.8)

Table 4 reports the association between anxiety and depression status and physical activity before and during quarantine in terms of OR of being borderline or being a severe case: compared to those who never exercised neither before nor during lockdown, the highest OR of being severely anxious or severely depressed was observed in those who stopped exercising (OR, 1.53; 95% CI, 1.28 to 1.84). Performing physical activity during lockdown was associated with a lower risk of being severely anxious or severely depressed in both groups: compared to those who never exercised, the odds for those who exercised during quarantine were 11% lower, although not statistically significant, if they were not physically active before quarantine, (OR, 0.89; 95% CI, 0.71 to 1.13) and 22% if they were physically active also before quarantine (OR, 0.78; 95% CI, 0.64 to 0.95). If during quarantine all individuals could continue to perform physical activity with the same frequency as before, the number of severe cases in the study population would have been 14% lower for anxiety (95% CI, 7 to 21%) and 21% lower for depression (95% CI, 10 to 32%). Similar results have been obtained for the two HADS subscales anxiety and depression separately (S2–S5 Tables).

**Table 4:** Effect of physical activity on psychological distress: Odds ratio of being borderline versus normal and severe versus normal.

		Level of Physical activity before lockdown	
		Less than one hour per week	More than one hour per week
		OR (95% CI)	OR (95% CI)
	**Level of Physical activity during lockdown**		
**Anxiety**			
**Borderline versus normal**			
	Less than 1 hour per week	Ref	1.20 (0.92 to 1.56)
	More than 1 hours per week	0.81 (0.66 to 1.00)	0.82 (0.69 to 0.97)
**Severe versus normal**			
	Less than 1 hour per week	Ref	1.52 (1.25 to 1.86)
	More than 1 hours per week	0.95 (0.74 to 1.23)	0.85 (0.71 to 1.03)
**Depression**			
**Borderline versus normal**			
	Less than 1 hour per week	Ref	1.39 (1.15 to 1.68)
	More than 1 hours per week	0.72 (0.53 to 0.97)	0.68 (0.57 to 0.81)
**Severe versus normal**			
	Less than 1 hour per week	Ref	1.71 (1.34 to 2.17)
	More than 1 hours per week	0.56 (0.41 to 0.75)	0.51 (0.40 to 0.65)
**Anxiety or depression**			
**Severe anxiety or severe depression versus normal**			
	Less than 1 hour per week	Ref	1.53 (1.28 to 1.84)
	More than 1 hours per week	0.89 (0.71 to 1.13)	0.78 (0.64 to 0.95)

## Discussion and conclusions

The results of this large cross-sectional study highlight the implications of quarantine on psychological distress in the adult population. Twenty percent of the participants reported severe levels of anxiety or depression. The factors associated with a higher level of psychological distress were being a student, having a low income or a low educational level within the family, having suffered of influenza-like symptoms or having a frail health, living with older or disabled people; all the observed associations were independent of the fear of being infected by the virus. Psychological distress was associated with distrust of the government and medical and scientific community; individuals reporting the higher level of distress were also those who believed that the measures implemented to contain the spreading of the virus were inadequate, either insufficient or too strict. With regard to physical activity, those who stopped exercising during quarantine had higher HADS scores. On the contrary, being able to perform some level of physical activity during quarantine was associated with lower HADS scores, irrespective of previous level of physical activity.

The proportion of participants in our survey reporting severe levels of anxiety or depression is similar to the proportions observed in other studies targeting European university communities: in a French and in a Spanish study, anxiety amounted to 27% and 21% and depression to 16% and 34%, respectively [12, 13].

In our population, those mostly affected by psychological distress were students and individuals with lower income, representing two groups of individuals highly suffering the occupational and economic uncertainty associated to the pandemic. Depression is known to be associated with the socio-economic status [21] and others reported that, during the COVID-19 pandemic as well as during other disasters, mental disorders were not evenly distributed in the population, being their incidence higher among disadvantaged groups of the population [22, 23].

The observed association between psychological distress and distrust of institutions, including government and medical staff, could be explained by the fact that people with an external locus of control—that is individuals believing that outcomes in their lives are determined by external factors—tend to cope worse with negative changes than those with an internal locus of control–that is individuals believing that outcomes in their lives depend largely on their own actions [24–29].

Stratifying our sample by level of physical activity before the lockdown period, we observed that subjects able to exercise during quarantine reported lower psychological distress irrespective of their previous level of physical activity. The association between level of physical activity during the lockdown period and more severe levels of anxiety and depression has been reported by others [16, 30–34]. One large cross-sectional study among French university students found association of low levels of physical activity with anxiety and depression [12]; one longitudinal study in the United Kingdom before and after lockdown showed positive association between change in perceived stress and change in sedentary behavior [10]. The evidence that mental health is associated with levels of physical activity independently of age and socio-economic status mainly comes from observational studies [15, 35] and clinical trials on the effect of exercise as treatment for depressions and anxiety [36–38]. Results from studies in animals and humans indicate that the effect of physical activity on depressive symptoms might be mediated through factors associated to monoamine metabolism and immune and neurogenic markers [39]. Not surprisingly, the cessation of physical activity has a negative impact on mental health [39, 40]. Accordingly, our data showed that those who stopped to exercise due to home confinement and closure of sport facilities experienced the most severe psychological distress. Although our results are in line with the literature supporting the hypothesis that reducing physical activity increases psychological distress, we cannot rule out a mechanism of reverse causality, resulting from a reduction of physical activity in the individuals more anxious or depressed.

The main strength of our study is its large sample size and the wide range of variables assessed, allowing to investigate the role of many potential determinants of psychological distress and to control for potential confounding. We targeted the entire university community, including students as well as the teaching, technical and administrative staff, in order to have access to a wide range of exposures and to be able to compare the conditions of students with those of the working individuals. The instrument used to assess the level of psychological distress was developed for hospitalized patients but is able to capture anxiety and depression symptoms in other contexts and samples. Indeed, the HADS proved to have good construct validity and other psychometric properties not only in clinical but also in general population samples [19, 20].

The representativeness of our sample at the level of the nationwide academic population is supported by the multicenter study design that we have adopted. However, because the survey has been completed on voluntary basis, we cannot exclude that a selection bias of participants based on their psychological distress levels might have occurred. However, there is no reason to believe that the potential selection might have affected the association between psychological distress and the predictors. Moreover, the large number of variables collected allowed to adjust the analyses for a wide range of potential confounders.

The negative impact of the COVID-19 pandemic on mental health is a matter of concern and experts urge for action to monitor and control its negative effects especially among most vulnerable individuals [41]. The knowledge accumulated from large epidemiological studies conducted during the first wave of COVID-19 can contribute to face future waves of infection or epidemic by identifying highly susceptible groups and possible intervention approaches. The study IO CONTO 2020, investigating the factors associated with anxiety and depression in a large sample of individuals, is placed in this perspective of action. Identifying economic instability among the factors associated to the increased risk of psychological impairment, we addressed once more the well-known consequences of social impairment on the health of a population. In the light of the finding that physical activity could at least partially relieve the psychological burden of a health crisis, we might suggest that future measures adopted to contain the spreading of a disease guarantee the access to outdoor spaces or safe indoor environment where citizens can perform their exercise. Further investigations are needed to quantify the long-term impact that COVID-19 quarantine had on the population.

## Supporting information

S1 Table Logistic regression of influenza-like symptoms during quarantine (temperature, cough or respiratory problems) or diagnosis of COVID-19. (DOCX)

S2 Table Linear regression of HADS-anxiety. (DOCX)

S3 Table Linear regression of HADS-depression. (DOCX)

S4 Table HADS-anxiety estimated marginal means (EMMs) by levels of physical activity. (DOCX)

S5 Table HADS-depression estimated marginal means (EMMs) by levels of physical activity. (DOCX)
