# Supplementary material for: Psychological distress in the academic population and its association with socio-demographic and lifestyle characteristics during COVID-19 pandemic lockdown: Results from a large multicenter Italian study
Source: PLoS One. 2021 Mar 10;16(3):e0248370. doi: 10.1371/journal.pone.0248370 (PMC7946293; doi:10.1371/journal.pone.0248370)
Supplement: S1 Table — (DOCX) [file pone.0248370.s001.docx]

**S1 Table.** Logistic regression of influenza-like symptoms during quarantine (temperature, cough or respiratory problems) or diagnosis of COVID-19.

|  | **Model 1** | | |  | **Model 2** | | |
| --- | --- | --- | --- | --- | --- | --- | --- |
| **Variable** | **Categorical** | **Pseudocontinuous** | |  | **Categorical** | **Pseudocontinuous** | |
|  | Coefficient (95% CI) | Coefficient (95% CI) | *P-value* |  | Coefficient (95% CI) | Coefficient (95% CI) | *P-value* |
| **Age (10-year increase)** | 0.89 (0.86 to 0.92) |  | *< 0.001* |  | 0.87 (0.80 to 0.95) |  | *0.001* |
| **Gender** | | | | | | | |
| Female | Ref |  | *0.006* |  | Ref |  | *0.02* |
| Male | 1.13 (1.04 to 1.23) |  |  |  | 1.19 (1.02 to 1.38) |  |  |
| **Position** | | | | | | | |
| Student | Ref | 0.92 (0.85 to 1.00) | *0.06* |  | Ref | 0.95 (0.86 to 1.06) | *0.38* |
| Technical/administrative staff | 0.68 (0.56 to 0.83) |  |  |  | 0.70 (0.55 to 0.90) |  |  |
| Teaching/research staff | 0.81 (0.68 to 0.98) |  |  |  | 0.88 (0.71 to 1.08) |  |  |
| **Income** | | | | | | | |
| Low | Ref | 0.95 (0.88 to 1.02) | *0.16* |  | Ref | 1.00 (0.92 to 1.08) | *0.94* |
| Medium | 0.92 (0.83 to 1.01) |  |  |  | 0.99 (0.88 to 1.11) |  |  |
| High | 0.89 (0.77 to 1.03) |  |  |  | 0.98 (0.83 to 1.16) |  |  |
| **Education level in the family** | | | | | | | |
| Primary | Ref | 0.99 (0.95 to 1.03) | *0.53* |  |  |  |  |
| Secondary | 0.96 (0.76 to 1.22) |  |  |  |  |  |  |
| University degree | 0.92 (0.65 to 1.31) |  |  |  |  |  |  |
| Master degree | 0.96 (0.68 to 1.35) |  |  |  |  |  |  |
| PhD or equivalent | 0.93 (0.68 to 1.28) |  |  |  |  |  |  |
| **House with a garden or balcony** | | | | | | | |
| No | Ref |  | *0.46* |  | Ref |  | *0.88* |
| Yes | 0.94 (0.80 to 1.10) |  |  |  | 0.99 (0.81 to 1.20) |  |  |
| **Cohabitants** |  |  |  |  |  |  |  |
| No | Ref |  | *0.85* |  |  |  |  |
| Yes | 1.02 (0.86 to 1.20) |  |  |  |  |  |  |
| **Old or disabled cohabitants** | | | | | | | |
| No | Ref |  | *0.42* |  | Ref |  | *0.45* |
| Yes | 1.04 (0.94 to 1.16) |  |  |  | 1.05 (0.92 to 1.21) |  |  |
| **Currently working with the public** | | | | | | | |
| No | Ref |  | *0.20* |  | Ref |  | *0.84* |
| Yes | 1.12 (0.94 to 1.33) |  |  |  | 1.02 (0.83 to 1.26) |  |  |
| **Cohabitants currently working with the public** | | | | | | | |
| No | Ref |  | *0.22* |  | Ref |  | *0.99* |
| Yes | 1.06 (0.97 to 1.15) |  |  |  | 1.00 (0.91 to 1.10) |  |  |
| **General health (number of comorbidities)** | | | | | | | |
| 0 | Ref | 1.61 (1.53 to 1.70) | *< 0.001* |  | Ref | 1.55 (1.43 to 1.67) | *< 0.001* |
| 1 | 1.72 (1.58 to 1.88) |  |  |  | 1.71 (1.54 to 1.91) |  |  |
| 2+ | 2.50 (2.22 to 2.82) |  |  |  | 2.19 (1.73 to 2.77) |  |  |
| **Worries** | | | | | | | |
| No | Ref |  | *< 0.001* |  | Ref |  | *0.008* |
| Yes | 1.33 (1.15 to 1.53) |  |  |  | 1.23 (1.06 to 1.42) |  |  |
| **Adequacy of the measures** | | | | | | | |
| Adequate | Ref | 1.18 (1.06 to 1.30) | *0.002* |  | Ref | 1.19 (1.04 to 1.37) | *0.01* |
| Insufficient | 1.29 (1.14 to 1.46) |  |  |  | 1.31 (1.11 to 1.53) |  |  |
| Excessive | 1.17 (0.98 to 1.40) |  |  |  | 1.22 (0.94 to 1.59) |  |  |
| **Trust in doctors** | | | | | | | |
| No | Ref |  | *0.26* |  |  |  |  |
| Yes | 0.90 (0.75 to 1.08) |  |  |  |  |  |  |
| **Trust in scientists** | | | | | | | |
| No | Ref |  | *0.06* |  |  |  |  |
| Yes | 0.84 (0.71 to 1.01) |  |  |  |  |  |  |
| **Trust in the government** | | | | | | | |
| No | Ref |  | *0.01* |  |  |  |  |
| Yes | 0.91 (0.84 to 0.98) |  |  |  |  |  |  |
| **Trust in doctors, scientists and the government** | | | | | | | |
| No | Ref |  | *0.004* |  | Ref |  | *0.64* |
| Yes | 0.90 (0.83 to 0.97) |  |  |  | 0.98 (0.88 to 1.08) |  |  |
| **Physical activity during quarantine** | | | | | | | |
| <1h | Ref | 0.89 (0.86 to 0.92) | *< 0.001* |  | Ref | 0.88 (0.84 to 0.92) | *< 0.001* |
| 1-2h | 0.94 (0.86 to 1.04) |  |  |  | 0.94 (0.84 to 1.07) |  |  |
| 3-4h | 0.77 (0.67 to 0.88) |  |  |  | 0.74 (0.65 to 0.84) |  |  |
| >4h | 0.72 (0.65 to 0.81) |  |  |  | 0.71 (0.62 to 0.81) |  |  |

Model 1: logistic regression adjusted for age and sex; Model 2: logistic regression adjusted for age and sex and all variables in the table. Model 2 is based on the subsample of 10,544 participants with no missing value in any of the variables in the table.
