# Supplementary material for: Psychological distress in the academic population and its association with socio-demographic and lifestyle characteristics during COVID-19 pandemic lockdown: Results from a large multicenter Italian study
Source: PLoS One. 2021 Mar 10;16(3):e0248370. doi: 10.1371/journal.pone.0248370 (PMC7946293; doi:10.1371/journal.pone.0248370)
Supplement: S2 Table — (DOCX) [file pone.0248370.s002.docx]

**S2 Table.** Linear regression of HADS-anxiety.

|  | **Model 1** | | |  | **Model 2** | | |
| --- | --- | --- | --- | --- | --- | --- | --- |
| **Variable** | **Categorical** | **Pseudocontinuous** | |  | **Categorical** | **Pseudocontinuous** | |
|  | Coefficient (95% CI) | Coefficient (95% CI) | *P-value* |  | Coefficient (95% CI) | Coefficient (95% CI) | *P-value* |
| **Age (10-year increase)** | -0.47 (-0.52 to -0.42) |  | *< 0.001* |  | -0.59 (-0.70 to -0.48) |  | *< 0.001* |
| **Gender** | | | | | | | |
| Female | Ref |  | *< 0.001* |  | Ref |  | *< 0.001* |
| Male | -1.91 (-2.06 to -1.76) |  |  |  | -1.70 (-1.90 to -1.51) |  |  |
| **Position** | | | | | | | |
| Student | Ref | -0.41 (-0.55 to -0.27) | *< 0.001* |  | Ref | -0.17 (-0.34 to 0.00) | *0.05* |
| Technical/administrative staff | -0.47 (-0.81 to -0.13) |  |  |  | -0.35 (-0.73 to 0.04) |  |  |
| Teaching/research staff | -0.84 (-1.12 to -0.55) |  |  |  | -0.38 (-0.71 to -0.04) |  |  |
| **Income** | | | | | | | |
| Low | Ref | -0.39 (-0.48 to -0.29) | *< 0.001* |  | Ref | -0.15 (-0.26 to -0.04) | *0.006* |
| Medium | -0.47 (-0.65 to -0.30) |  |  |  | -0.08 (-0.33 to 0.17) |  |  |
| High | -0.78 (-0.97 to -0.59) |  |  |  | -0.30 (-0.51 to -0.08) |  |  |
| **Education level in the family** | | | | | | | |
| Primary | Ref | -0.17 (-0.23 to -0.11) | *< 0.001* |  |  |  |  |
| Secondary | -0.49 (-0.91 to -0.07) |  |  |  |  |  |  |
| University degree | -0.75 (-1.19 to -0.31) |  |  |  |  |  |  |
| Master degree | -0.52 (-1.00 to -0.04) |  |  |  |  |  |  |
| PhD or equivalent | -1.01 (-1.52 to -0.50) |  |  |  |  |  |  |
| **House with a garden or balcony** | | | | | | | |
| No | Ref |  | *< 0.001* |  | Ref |  | *< 0.001* |
| Yes | -0.89 (-1.16 to -0.63) |  |  |  | -0.81 (-1.05 to -0.57) |  |  |
| **Cohabitants** |  |  |  |  |  |  |  |
| No | Ref |  | *0.91* |  |  |  |  |
| Yes | -0.02 (-0.43 to 0.38) |  |  |  |  |  |  |
| **Old or disabled cohabitants** | | | | | | | |
| No | Ref |  | *< 0.001* |  | Ref |  | *0.02* |
| Yes | 0.50 (0.23 to 0.78) |  |  |  | 0.25 (0.03 to 0.46) |  |  |
| **Currently working with the public** | | | | | | | |
| No | Ref |  | *0.008* |  | Ref |  | *0.06* |
| Yes | -0.41 (-0.72 to -0.11) |  |  |  | -0.50 (-1.01 to 0.01) |  |  |
| **Cohabitants currently working with the public** | | | | | | | |
| No | Ref |  | *< 0.001* |  | Ref |  | *< 0.001* |
| Yes | 0.26 (0.12 to 0.40) |  |  |  | 0.30 (0.14 to 0.46) |  |  |
| **General health (number of comorbidities)** | | | | | | | |
| 0 | Ref | 1.20 (0.98 to 1.43) | *< 0.001* |  | Ref | 0.97 (0.72 to 1.21) | *< 0.001* |
| 1 | 1.14 (0.89 to 1.39) |  |  |  | 1.00 (0.66 to 1.35) |  |  |
| 2+ | 2.46 (1.99 to 2.93) |  |  |  | 1.91 (1.48 to 2.34) |  |  |
| **Symptoms** | | | | | | | |
| No | Ref |  | *< 0.001* |  | Ref |  | *< 0.001* |
| Yes | 1.09 (0.91 to 1.27) |  |  |  | 0.74 (0.51 to 0.97) |  |  |
| **Worries** | | | | | | | |
| No | Ref |  | *< 0.001* |  | Ref |  | *< 0.001* |
| Yes | 1.90 (1.69 to 2.12) |  |  |  | 1.63 (1.42 to 1.84) |  |  |
| **Adequacy of the measures** | | | | | | | |
| Adequate | Ref | 0.85 (0.74 to 0.95) | *< 0.001* |  | Ref | 0.53 (0.39 to 0.66) | *< 0.001* |
| Insufficient | 0.99 (0.86 to 1.13) |  |  |  | 0.58 (0.42 to 0.75) |  |  |
| Excessive | 1.31 (0.93 to 1.70) |  |  |  | 0.87 (0.35 to 1.39) |  |  |
| **Trust in doctors** | | | | | | | |
| No | Ref |  | *< 0.001* |  |  |  |  |
| Yes | -1.51 (-2.05 to -0.98) |  |  |  |  |  |  |
| **Trust in scientists** | | | | | | | |
| No | Ref |  | *< 0.001* |  |  |  |  |
| Yes | -1.27 (-1.98 to -0.55) |  |  |  |  |  |  |
| **Trust in the government** | | | | | | | |
| No | Ref |  | *< 0.001* |  |  |  |  |
| Yes | -0.92 (-1.12 to -0.71) |  |  |  |  |  |  |
| **Trust in doctors, scientists and the government** | | | | | | | |
| No | Ref |  | *< 0.001* |  | Ref |  | *< 0.001* |
| Yes | -0.94 (-1.15 to -0.73) |  |  |  | -0.66 (-0.87 to -0.45) |  |  |
| **Physical activity during quarantine** | | | | | | | |
| <1h | Ref | -0.32 (-0.38 to -0.25) | *< 0.001* |  | Ref | -0.27 (-0.34 to -0.20) | *< 0.001* |
| 1-2h | -0.50 (-0.69 to -0.31) |  |  |  | -0.39 (-0.60 to -0.17) |  |  |
| 3-4h | -0.89 (-1.07 to -0.72) |  |  |  | -0.72 (-0.93 to -0.51) |  |  |
| >4h | -0.85 (-1.09 to -0.61) |  |  |  | -0.74 (-0.96 to -0.52) |  |  |

Model 1: linear regression adjusted for age and sex; Model 2: linear regression adjusted for age and sex and all variables in the table. Model 2 is based on the subsample of 9,970 participants with no missing value in any of the variables in the table.
