# Supplementary material for: Psychological distress in the academic population and its association with socio-demographic and lifestyle characteristics during COVID-19 pandemic lockdown: Results from a large multicenter Italian study
Source: PLoS One. 2021 Mar 10;16(3):e0248370. doi: 10.1371/journal.pone.0248370 (PMC7946293; doi:10.1371/journal.pone.0248370)
Supplement: S3 Table — (DOCX) [file pone.0248370.s003.docx]

**S3 Table.** Linear regression of HADS-depression.

|  | **Model 1** | | |  | **Model 2** | | |
| --- | --- | --- | --- | --- | --- | --- | --- |
| **Variable** | **Categorical** | **Pseudocontinuous** | |  | **Categorical** | **Pseudocontinuous** | |
|  | Coefficient (95% CI) | Coefficient (95% CI) | *P-value* |  | Coefficient (95% CI) | Coefficient (95% CI) | *P-value* |
| **Age (10-year increase)** | -0.38 (-0.46 to -0.30) |  | *< 0.001* |  | -0.54 (-0.64 to -0.44) |  | *< 0.001* |
| **Gender** | | | | | | | |
| Female | Ref |  | *< 0.001* |  | Ref |  | *< 0.001* |
| Male | -0.79 (-0.91 to -0.67) |  |  |  | -0.74 (-0.89 to -0.59) |  |  |
| **Position** | | | | | | | |
| Student | Ref | -0.37 (-0.50 to -0.24) | *< 0.001* |  | Ref | -0.19 (-0.34 to -0.05) | *0.009* |
| Technical/administrative staff | -0.41 (-0.70 to -0.11) |  |  |  | -0.27 (-0.61 to 0.07) |  |  |
| Teaching/research staff | -0.74 (-1.00 to -0.48) |  |  |  | -0.42 (-0.71 to -0.12) |  |  |
| **Income** | | | | | | | |
| Low | Ref | -0.34 (-0.42 to -0.25) | *< 0.001* |  | Ref | -0.11 (-0.20 to -0.01) | *0.03* |
| Medium | -0.43 (-0.59 to -0.28) |  |  |  | -0.14 (-0.31 to 0.04) |  |  |
| High | -0.68 (-0.85 to -0.51) |  |  |  | -0.21 (-0.41 to -0.02) |  |  |
| **Education level in the family** | | | | | | | |
| Primary | Ref | -0.14 (-0.21 to -0.06) | *< 0.001* |  |  |  |  |
| Secondary | -0.12 (-0.62 to 0.38) |  |  |  |  |  |  |
| University degree | -0.27 (-0.74 to 0.20) |  |  |  |  |  |  |
| Master degree | -0.23 (-0.96 to 0.49) |  |  |  |  |  |  |
| PhD or equivalent | -0.56 (-1.15 to 0.02) |  |  |  |  |  |  |
| **House with a garden or balcony** | | | | | | | |
| No | Ref |  | *< 0.001* |  | Ref |  | *< 0.001* |
| Yes | -0.74 (-0.98 to -0.49) |  |  |  | -0.52 (-0.73 to -0.30) |  |  |
| **Cohabitants** |  |  |  |  |  |  |  |
| No | Ref |  | *0.51* |  |  |  |  |
| Yes | -0.19 (-0.74 to 0.37) |  |  |  |  |  |  |
| **Old or disabled cohabitants** | | | | | | | |
| No | Ref |  | *< 0.001* |  | Ref |  | *0.05* |
| Yes | 0.39 (0.23 to 0.55) |  |  |  | 0.19 (0.00 to 0.38) |  |  |
| **Currently working with the public** | | | | | | | |
| No | Ref |  | *< 0.001* |  | Ref |  | *< 0.001* |
| Yes | -0.56 (-0.83 to -0.29) |  |  |  | -0.74 (-1.15 to -0.33) |  |  |
| **Cohabitants currently working with the public** | | | | | | | |
| No | Ref |  | *0.10* |  | Ref |  | *0.04* |
| Yes | 0.10 (-0.02 to 0.22) |  |  |  | 0.18 (0.01 to 0.34) |  |  |
| **General health (number of comorbidities)** | | | | | | | |
| 0 | Ref | 1.02 (0.82 to 1.21) | *< 0.001* |  | Ref | 0.83 (0.60 to 1.06) | *< 0.001* |
| 1 | 0.93 (0.69 to 1.17) |  |  |  | 0.87 (0.53 to 1.20) |  |  |
| 2+ | 2.08 (1.71 to 2.45) |  |  |  | 1.62 (1.23 to 2.01) |  |  |
| **Symptoms** | | | | | | | |
| No | Ref |  | *< 0.001* |  | Ref |  | *< 0.001* |
| Yes | 0.81 (0.64 to 0.98) |  |  |  | 0.46 (0.22 to 0.70) |  |  |
| **Worries** | | | | | | | |
| No | Ref |  | *< 0.001* |  | Ref |  | *< 0.001* |
| Yes | 0.87 (0.72 to 1.02) |  |  |  | 0.69 (0.52 to 0.86) |  |  |
| **Adequacy of the measures** | | | | | | | |
| Adequate | Ref | 0.77 (0.67 to 0.87) | *< 0.001* |  | Ref | 0.59 (0.47 to 0.71) | *< 0.001* |
| Insufficient | 0.66 (0.54 to 0.78) |  |  |  | 0.47 (0.33 to 0.62) |  |  |
| Excessive | 1.78 (1.41 to 2.15) |  |  |  | 1.43 (1.01 to 1.84) |  |  |
| **Trust in doctors** | | | | | | | |
| No | Ref |  | *< 0.001* |  |  |  |  |
| Yes | -1.73 (-2.01 to -1.44) |  |  |  |  |  |  |
| **Trust in scientists** | | | | | | | |
| No | Ref |  | *< 0.001* |  |  |  |  |
| Yes | -1.33 (-1.62 to -1.04) |  |  |  |  |  |  |
| **Trust in the government** | | | | | | | |
| No | Ref |  | *< 0.001* |  |  |  |  |
| Yes | -0.90 (-1.02 to -0.78) |  |  |  |  |  |  |
| **Trust in doctors, scientists and the government** | | | | | | | |
| No | Ref |  | *< 0.001* |  | Ref |  | *< 0.001* |
| Yes | -0.92 (-1.05 to -0.79) |  |  |  | -0.65 (-0.82 to -0.49) |  |  |
| **Physical activity during quarantine** | | | | | | | |
| <1h | Ref | -0.60 (-0.65 to -0.55) | *< 0.001* |  | Ref | -0.56 (-0.62 to -0.5) | *< 0.001* |
| 1-2h | -0.99 (-1.14 to -0.84) |  |  |  | -0.86 (-1.04 to -0.68) |  |  |
| 3-4h | -1.48 (-1.63 to -1.33) |  |  |  | -1.32 (-1.50 to -1.13) |  |  |
| >4h | -1.70 (-1.86 to -1.54) |  |  |  | -1.63 (-1.82 to -1.43) |  |  |

Model 1: linear regression adjusted for age and sex; Model 2: linear regression adjusted for age and sex and all variables in the table. Model 2 is based on the subsample of 9,385 participants with no missing value in any of the variables in the table.
