# Supplementary material for: Psychological distress in the academic population and its association with socio-demographic and lifestyle characteristics during COVID-19 pandemic lockdown: Results from a large multicenter Italian study
Source: PLoS One. 2021 Mar 10;16(3):e0248370. doi: 10.1371/journal.pone.0248370 (PMC7946293; doi:10.1371/journal.pone.0248370)
Supplement: S4 Table — (DOCX) [file pone.0248370.s004.docx]

**S4 Table.** HADS-anxiety estimated marginal means (EMMs) by levels of physical activity.

|  | **Physical activity before lockdown** | | |
| --- | --- | --- | --- |
|  | Less than one hour per week |  | At least one our per week |
|  | EMM (95% CI) |  | EMM (95% CI) |
| **Physical activity during lockdown** | | | |
| Less than 1 hour per week | 6.9 (6.7 to 7.1) |  | 7.5 (7.2 to 7.8) |
| 1-2 hours per week | 6.9 (6.4 to 7.4) |  | 6.8 (6.5 to 7.2) |
| 3-4 hours per week | 6.5 (6.1 to 6.9) |  | 6.5 (6.3 to 6.8) |
| More than 4 hours per week | 6.5 (5.9 to 7.1) |  | 6.5 (6.3 to 6.8) |
